# Manifestations of Fasting-Induced Fatty Liver and Rapid Recovery from Steatosis in Voles Fed Lard or Flaxseed Oil Lipids

**DOI:** 10.3390/nu5104211

**Published:** 2013-10-22

**Authors:** Anne-Mari Mustonen, Vesa Kärjä, Michael Kilpiö, Raija Tammi, Markku Tammi, Kirsti Rouvinen-Watt, Toivo Halonen, Petteri Nieminen

**Affiliations:** 1 Faculty of Health Sciences, Institute of Biomedicine/Anatomy, School of Medicine, University of Eastern Finland, P.O. Box 1627, Kuopio FI-70211, Finland; E-Mails: mikilpio@student.uef.fi (M.K.); raija.tammi@uef.fi (R.T.); markku.tammi@uef.fi (M.T.); petteri.nieminen@uef.fi (P.N.); 2 Faculty of Science and Forestry, Department of Biology, University of Eastern Finland, P.O. Box 111, Joensuu FI-80101, Finland; 3 Department of Clinical Pathology, Kuopio University Hospital, P.O. Box 1777, Kuopio FI-70211, Finland; E-Mail: vesa.karja@gmail.com; 4 Faculty of Agriculture, Department of Plant and Animal Sciences, Dalhousie University, P.O. Box 550, Truro, NS B2N 5E3, Canada; E-Mail: kirsti.rouvinen-watt@dal.ca; 5 Eastern Finland Laboratory Centre (ISLAB), P.O. Box 1700, Kuopio FI-70211, Finland; E-Mail: toivo.halonen@islab.fi

**Keywords:** fasting, fatty liver, flaxseed oil, hyaluronan, lard, α-linolenic acid, *Microtus* voles, *n*-3 PUFA, SFA, steatosis

## Abstract

Long-chain *n*-3 polyunsaturated fatty acids (PUFA) can have beneficial effects against fat deposition, cardiovascular diseases, and liver steatosis. We investigated how diets based on lard (predominantly saturated and monounsaturated fatty acids) or flaxseed oil (rich in 18:3*n*-3) affect liver fat-% and fatty acid profiles of tundra voles (*Microtus oeconomus*). We also studied potential participation of hyaluronan (HA) in the pathology of fatty liver and whether the development and recovery of fasting-induced steatosis are influenced by *n*-3 PUFA. The dietary fatty acid composition was manifested in the liver fatty acid signatures. Fasting for 18 h induced macrovesicular steatosis and the liver fat-% increased to 22% independent of the preceding diet. Fasting-induced steatosis did not involve inflammation or connective tissue activation indicated by the absence of both leukocyte accumulation and increased HA. Food deprivation modified the liver fatty acid signatures to resemble more closely the diets. Fasting reduced the proportions of long-chain *n*-3 PUFA in both dietary regimes and *n*-3/*n*-6 PUFA ratios in the lard-fed voles. Decreases in long-chain *n*-3 PUFA may promote lipid accumulation by modulating the expression of lipid-metabolizing genes. Dietary 18:3*n*-3 did not prevent the development or attenuate the manifestation of steatosis in the fasted voles or promote the recovery.

## 1. Introduction

Non-alcoholic fatty liver disease (NAFLD) is a major health burden in Western countries, affecting 20%–30% of the general population [1,2]. While NAFLD is frequently asymptomatic and relatively benign, it can develop into non-alcoholic steatohepatitis (NASH), which has the potential to progress to cirrhosis and hepatocarcinoma. NAFLD is characterized by pathological accumulation of lipids in the liver in the absence of alcohol abuse, but its pathogenesis is still inadequately understood. Generally, fat accumulation arises from an imbalance between hepatic lipid uptake, oxidation, export of lipids from the liver, and *de novo* lipogenesis [3]. This has been considered the first hit in NAFLD predisposing the liver to injury mediated by second hits, such as inflammatory cytokines/adipokines, mitochondrial dysfunction, and oxidative stress, leading to NASH and fibrosis [4].

NAFLD is closely associated with features of the metabolic syndrome, such as obesity, insulin resistance, hyperlipidemia, and hypertension, but can also occur in starvation [1,2]. Its pathogenesis may be influenced by the hepatic fatty acid (FA) profile [5]. In general, the dietary *n*-3/*n*-6 polyunsaturated FA (PUFA) ratio should be approximately 1:3, but the modern diet is rich in *n*-6 PUFA and, in some cases, the ratio can be close to 1:15 [2]. This affects the *n*-3/*n*-6 PUFA balance of the body and the nature of eicosanoids synthesized from their precursors 20:5*n*-3 (EPA) and 20:4*n*-6 (ARA), as *n*-3 and *n*-6 PUFA are competitively metabolized by the same pathway [6]. Generally, ARA-derived eicosanoids are proinflammatory and prothrombotic and those from EPA anti-inflammatory and antithrombotic. In reference to hepatology, NAFLD is characterized by a depletion of long-chain (LC) *n*-3 and *n*-6 PUFA (ARA, EPA, and 22:6*n*-3 [DHA]) from the liver and by decreased liver and adipose tissue *n*-3/*n*-6 PUFA ratios [5,7].

Diets with high levels of saturated FA (SFA) and sucrose and those containing *t*10, *c*12 conjugated linoleic acid (CLA) were steatogenic in rodents [8,9,10], and high SFA intake promoted the progression of NASH to fibrosis [11]. NAFLD patients can consume less fish and more meat compared to subjects with normal livers [12]. Furthermore, the diet of NASH patients was poor in PUFA and fiber, but rich in SFA and cholesterol [13] and, according to another study, their diet contained less carbohydrates and protein and more total fat, monounsaturated FA (MUFA), and *n*-6 PUFA with a lower *n*-3/*n*-6 PUFA ratio [14]. Liver fibrosis and inflammation have been observed to be concomitant with higher serum hyaluronan (hyaluronic acid, HA) concentrations [15], and it has been established that diabetic patients with fatty liver disease have higher HA levels in circulation than non-diabetics [16]. HA increases rapidly in diverse inflammatory states such as in bacterial and viral infections, autoimmune inflammation, and metabolic derangements, as well as in conditions with connective tissue activation due to various stimuli [17,18,19].

At present, there is no consensus on the possible nutritional or medical treatments of NAFLD [2]. In addition to dietary changes, weight loss, exercise, and treatment of coexisting disorders, recent studies on animal models suggest that *n*-3 PUFA supplements with EPA and DHA could be promising remedies to reduce hepatic steatosis, improve insulin sensitivity, and decrease markers of inflammation and oxidative stress. For example, the oral administration of EPA ameliorated hepatic fat accumulation in mice (*Mus musculus*) fed a Western-style diet by downregulating the expression of several lipogenic genes [8]. LC *n*-3 PUFA could also increase FA oxidation by activating the transcription factor peroxisome proliferator-activated receptor α [20,21]. In NAFLD patients, prolonged *n*-3 PUFA supplementation improved insulin sensitivity and ultrasonographic features of the livers [22,23,24].

*Microtus* voles represent attractive model species as they experience pronounced accumulation of liver triacylglycerols (TAG) when fasted for only 4–18 h [25,26], the resulting steatosis resembling that of NAFLD patients despite of different etiologies [5,26]. Also, a high-fat diet can induce hepatic fatty infiltration to voles that, in addition to this, are susceptible to diet-induced atherosclerosis [27,28]. The diets selected for the present study were based on lard (predominantly SFA and MUFA) or flaxseed oil lipids (α-linolenic acid [ALA]). Lard-based diets are known to be steatogenic in murine models [29], while flaxseed oil can attenuate non-alcoholic fatty liver in hyperlipidemic rodents [30] and reverse some adverse metabolic effects of CLA including insulin resistance and development of fatty liver [9]. In addition, in poultry, diets with flaxseed decrease liver fat content while lard diets promote the opposite effect [31,32]. The specific aims of this study were to examine: (i) how diets based on lard or flaxseed oil affect the food intake, body fat stores, liver fat-%, and FA profiles of voles, (ii) whether the different diets could have effects on the rate of weight loss and the responses of liver biochemistry and histology to 18 h of fasting, (iii) if signs of inflammation and connective tissue activation (leukocytes and HA accumulation) are present in steatotic livers of voles, and (iv) how body composition and liver characteristics recover from food deprivation. Based on previous data, it was hypothesized that flaxseed oil *n*-3 PUFA (dietary ALA and its derivatives EPA and DHA) could have beneficial effects on the prevention of fasting-induced fatty liver or on the recovery from steatosis.

## 2. Experimental Section

The tundra voles *Microtus oeconomus* (*n* = 40) were obtained from the laboratory colony of the University of Eastern Finland. They were maintained in a dark room with illumination from 0700 to 2100 h (14L:10D) at 20 ± 1 °C and housed singly in solid-bottomed plastic cages (42 × 22 × 15 cm) with wood shavings for bedding and free access to water and a pelleted diet (Avelsfoder för råtta och mus R36; 18.5% protein, 4.0% fat, 55.7% carbohydrates, 301 kcal metabolizable energy/100 g, Lactamin, Stockholm, Sweden). The experiment was licensed by the Finnish National Animal Experiment Board (decision number ESLH-2009-08219/Ym-23, date of approval 10/28/2009) and complied with the current laws of Finland.

The voles were divided into two groups that were fed for eight weeks with two different experimental diets (Harlan Laboratories, Madison, WI, USA; Table 1). The composition of the diets was otherwise similar, but Diet 1 contained 6.5% lard as the primary source of lipids (Lard voles), while Diet 2 contained 6.5% flaxseed oil (Flaxseed voles) yielding clearly divergent dietary FA profiles. Both diets were supplemented with soybean oil (1.5%) to ensure the availability of essential ALA to the lard-fed voles. The diets were stored at −50 °C and only the required portion was thawed weekly. After eight weeks, both dietary groups were divided into four study groups of five animals (two to three males and two to three females; Table 2) as follows: fed control voles (Lard C, Flax C); voles food-deprived for 18 h (Lard F, Flax F); voles food-deprived for 18 h and refed for 24 h (Lard F+RF1d, Flax F+RF1d); and voles food-deprived for 18 h and refed for seven days (Lard F+RF7d, Flax F+RF7d). Refeeding for one or seven days (1dRF, 7dRF) was conducted with the same diets used prior to the fasting period.

**Table 1:** Composition of the experimental diets.

Formula (g/kg)	Lard Diet	Flaxseed Oil Diet
Casein	150	150
l-Cystine	2.25	2.25
Corn starch	260.2	260.2
Maltodextrin	80	80
Sucrose	100	100
Lard	65	–
Flaxseed oil	–	65
Soybean oil	15	15
Cellulose	270	270
Mineral mix, w/o Ca & P	23	23
Calcium carbonate	7.5	7.5
Calcium phosphate	17	17
Vitamin mix	10	10
Antioxidant (TBHQ)	0.014	0.014
**Nutrient Information (wt-%)**		
Protein	13.7	13.7
Carbohydrate	41.4	41.4
Fat	8.0	8.0
Kcal/g	2.9	2.9

TBHQ = *tert*-butylhydroquinone.

**Table 2:** Abbreviations of the experimental groups.

Principal Fat Source in Feed	Assignment of Voles to Study Groups (*n* = 5)	Group Abbreviation
Lard (*n* = 20)	Fed controls	Lard C
	Fasted for 18 h	Lard F
	Fasted for 18 h and refed for 1 day	Lard F+RF1d
	Fasted for 18 h and refed for 7 days	Lard F+RF7d
Flaxseed oil (*n* = 20)	Fed controls	Flax C
	Fasted for 18 h	Flax F
	Fasted for 18 h and refed for 1 day	Flax F+RF1d
	Fasted for 18 h and refed for 7 days	Flax F+RF7d

The body masses (BM) were measured at the beginning of the experimental feeding, at the onset and end of the food deprivation period as well as after refeeding. There were no significant differences in the initial BM (average of all voles: 31.9 ± 0.78 g) or ages (185 ± 3 days) between the study groups. The food intake was measured weekly, when the uneaten food was weighed and replaced with fresh pellets. For sampling, the animals were sacrificed between 0800 and 1000 h with an overdose of diethyl ether. Livers were dissected, weighed, frozen in liquid nitrogen, and stored at −70 °C. The visible adipose tissue depots were dissected and weighed.

Subsamples of the diets and livers were transmethylated [33], and the formed FA methyl esters were extracted with hexane and analyzed by an Agilent 6850 gas–liquid chromatograph (Agilent Technologies, Santa Clara, CA, USA; [26]). The results represent the FA composition (mol-%) of the total lipids. The hepatic TAG and cholesterol concentrations were determined [34] and the liver lipids extracted for fat-% calculations as outlined previously [35]. Fractionation coefficients between the dietary and tissue levels of each FA were calculated as: (mol-% in tissue)/(average mol-% in diet).

A portion of each fresh liver was stored in neutral formalin fixative, dehydrated and embedded in paraffin, cut into sections, and attached to glass slides. After staining with hematoxylin–eosin, the slides were examined three times at 400× magnification under a light microscope (Leica DM LB, Leica Microsystems, Heerbrugg, Switzerland) by a consultant pathologist (V. Kärjä) in a randomized double-blind manner. The criteria by Kleiner *et al*. [36] were used to evaluate the histological appearance. Briefly, the samples were examined for macro- and microvesicular steatosis and fibrosis. The extent of steatosis was graded based on the percentage of hepatocytes with this type of pathology (<5%, 5%–33%, >33%–66%, and >66%; classified as 0–3). Inflammatory changes were assessed by observing the presence of lobular and portal inflammation and micro- and lipogranulomas. Liver cell injury was determined by observing ballooning and the presence of acidophil bodies, pigmented macrophages, and megamitochondria. The samples were also examined for Mallory’s hyaline and glycogenated nuclei and evaluated for the presence of borderline or definite NASH.

The paraffin-embedded liver samples were also processed for HA detection [37]. The sections were incubated with 1% H_2_O_2_ for five minutes to block tissue peroxidase activity. Thereafter, they were incubated with 1% bovine serum albumin in phosphate buffer (PB) for 30 min at 37 °C, followed by overnight incubation at 4 °C with 3 µg/mL of biotinylated HA-binding complex, isolated from calf articular cartilage and containing the link protein and G1 domain of aggrecan. After washes with PB, the sections were incubated with avidin–biotin peroxidase (Vector Laboratories, Burlingame, CA, 1:200) for one hour. The color was developed with 0.05% 3,3′-diaminobenzidine (Sigma-Aldrich, St. Louis, MO, USA) containing 0.03% H_2_O_2_. The sections were counterstained with Mayer’s hematoxylin for two minutes, washed, dehydrated, and mounted in DePex (BDH Laboratory Supplies, Poole, UK). The presence of HA was evaluated by the consultant pathologist by estimating the extent of portal field reactivity (range 0–3) in basement membrane and connective tissues around bile ducts and in venous endothelium.

Multiple comparisons within a diet were performed with the oneway analysis of variance (ANOVA) followed by the *post hoc* Duncan’s test or, in the case of nonparametric variables, with the Kruskal–Wallis ANOVA and the *post hoc* Dunn’s test (SPSS v19 software package, IBM, Armonk, NY, USA). Comparisons of similar treatments between diets were performed with the Student’s *t-*test or with the Mann–Whitney U test for nonparametric data. Changes in the BM were compared with the repeated measures ANOVA, histological data were tested with the Kruskal–Wallis exact test, and correlations calculated with the Spearman correlation coefficient (*r*_s_). The *p* value < 0.05 was considered statistically significant. The results are presented as the mean ± SE.

## 3. Results

### 3.1. BM Changes and Food Intake

The BM of all study groups increased during the initial 8-week feeding period (repeated measures ANOVA, *p* < 0.001). There were no significant differences in the BM gain between the dietary groups (Lard voles: 17.6% ± 2.6%; Flaxseed voles: 24.0% ± 2.8%). Fasting for 18 h caused a decrease in the BM (repeated measures ANOVA, *p* < 0.001), but the preceding diets did not affect the rate of weight loss (10.1% ± 0.6% for all fasted voles). In addition, the BM gains after fasting were significant (repeated measures ANOVA, *p* < 0.001) but unaffected by the fat source (1dRF groups: 4.5% ± 1.5%; 7dRF groups: 4.5% ± 1.0%). Nor were there any diet-induced differences in the food intake before (Lard voles: 10.0 ± 0.42 g feed/g BM; Flaxseed voles: 10.6 ± 0.54 g feed/g BM) or after food deprivation (1dRF voles: 0.24 ± 0.031 *vs.* 0.25 ± 0.030 g feed/g BM; 7dRF voles: 1.5 ± 0.11 *vs.* 1.7 ± 0.17 g feed/g BM).

### 3.2. Liver Weights, Fat-%, TAG and Cholesterol Concentrations

**Figure 1:**
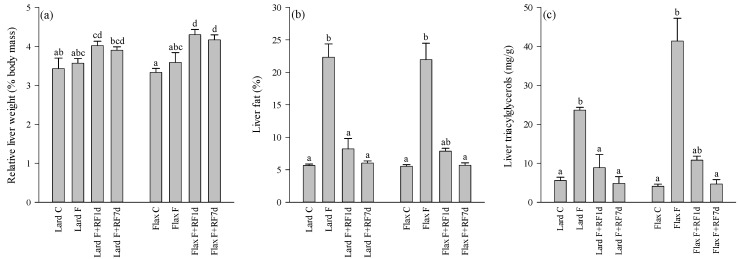
The relative liver weights (% body mass; **a**), hepatic fat-% (**b**), and triacylglycerol concentrations (mg/g; **c**) of the tundra voles fed lard- or flaxseed oil-based diets, fasted for 18 h, and refed for 1 or 7 days after the 18-h fast (mean + SE). Means with no common letters are statistically different from each other within a diet (oneway ANOVA, Kruskal–Wallis ANOVA, *p* < 0.05). Abbreviations of the study groups are explained in Table 2.

The absolute liver weights were higher in the Flax F+RF7d group compared to the Flax C voles (oneway ANOVA, *p* < 0.05; data not shown). The same was observed in the relative liver masses of the Lard F+RF1d, Flax F+RF1d, and Flax F+RF7d groups compared to their controls (oneway ANOVA, *p* < 0.001; Figure 1a). The liver fat-% correlated inversely with the hepatic 18:0 (*r*_s_ = −0.831, *p* < 0.001) and ARA percentages (*r*_s_ = −0.718, *p* < 0.001). There were no differences in the masses of specific fat depots or body fat-% between the groups. The averages for intra-abdominal and subcutaneous fat masses of all animals were 1.0 ± 0.10 g and 3.3 ± 0.30 g, respectively, and based on these data, the mean body fat-% was calculated to be 11.2 ± 0.83%.

The liver fat-% averaged 5.6 ± 0.15% in the fed animals and increased to 22.1 ± 1.53% at 18 h of fasting, with no effects of the dietary fat source (Kruskal–Wallis ANOVA, *p* < 0.001; Figure 1b). The values returned close to the control levels after one day of refeeding. Similar patterns of change were observed for the hepatic TAG (Figure 1c) and cholesterol concentrations (Kruskal–Wallis ANOVA, *p* < 0.001).

### 3.3. Liver Histology

**Figure 2:**
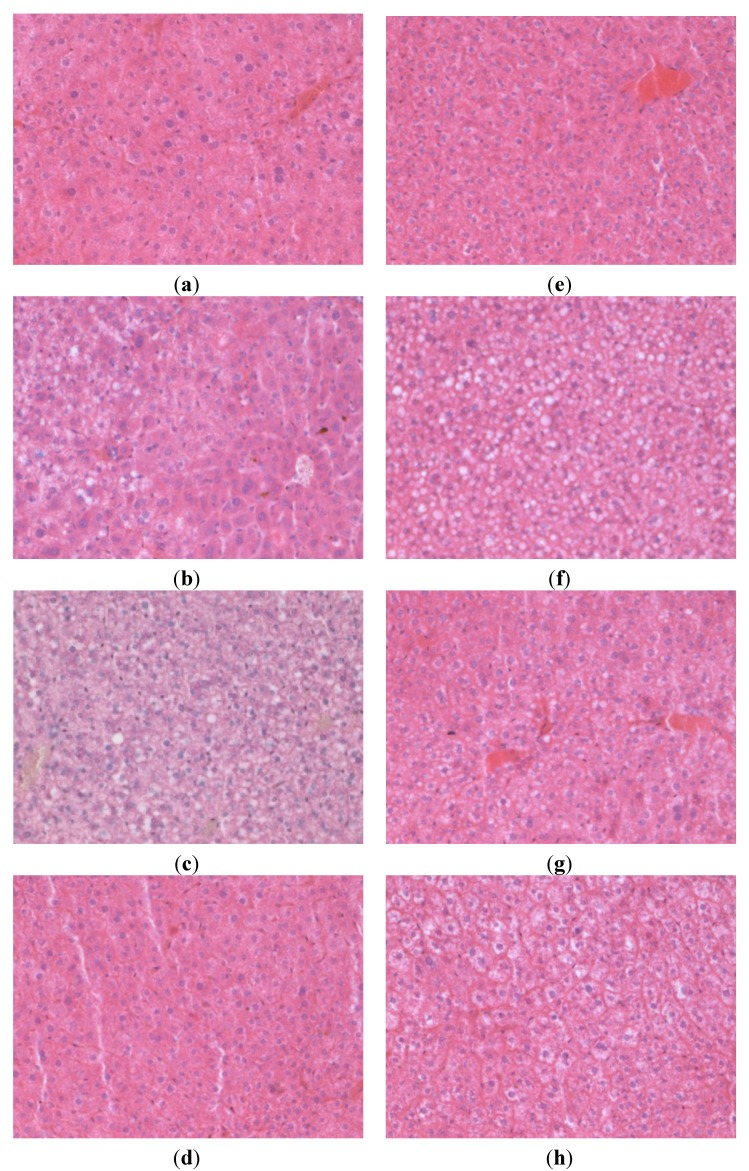
Microscopic images of hematoxylin–eosin-stained liver tissue (200× magnification) from tundra voles representing the different study groups: (**a**) Lard C, (**b**) Lard F, (**c**) Lard F+RF1d, (**d**) Lard F+RF7d, (**e**) Flax C, (**f**) Flax F, (**g**) Flax F+RF1d, (**h**) Flax F+RF7d. Abbreviations of the study groups are explained in Table 2.

Fasting increased the occurrence of (primarily macrovesicular) steatosis in the voles (Kruskal–Wallis exact test, *p* < 0.001), but the diets had no influence (Figure 2, Table 3). In the lard-fed control animals, 2/5 individuals had grade 1–2 fatty liver, while 5/5 of the fasted animals had grades 2–3. In the 1d-refed group, 2/5 individuals had grade 1 steatosis. After seven days of refeeding, none of the lard-fed animals showed fatty liver. No flaxseed oil-fed controls had fatty liver, 5/5 of the fasted animals showed grade 3 steatosis, 2/5 of the refed individuals had grade 1–2 steatosis after one day, and there were no individuals with fatty livers after seven days of refeeding. The location of lipid droplets was panacinar (6 cases), azonal (5 cases), zone 3 (3 cases), and zone 1 (2 cases) without any diet-induced effects on the distribution of steatosis. Ballooning degeneration (indicating liver damage) and hepatic regeneration (mitoses and hypertrophic nuclei indicating liver recovery) were observed in 10 and 7 refed voles, respectively, mainly after one day of refeeding in both dietary regimes. There were only three cases of inflammation indicated by the presence of neutrophils, and none of the animals showed fibrosis. The localization and extent of HA did not differ between the principal fat sources or according to the fasting and refeeding treatments (Table 4). While many animals displayed HA staining, these findings were distributed also among the voles with no signs of liver inflammation.

**Table 3:** Occurrence of primarily macrovesicular steatosis (range 0–3 according to the extent of lipidosis) and distribution of lipid droplets in the tundra vole livers.

	Steatosis Grading				Distribution			
Study Group	0	1	2	3	Zone 1	Zone 3	Panacinar	Azonal
Lard C	3	1	1		1			1
Lard F			2	3	1	2	2	
Lard F+RF1d	3	2						2
Lard F+RF7d	5							
Flax C	5							
Flax F				5		1	4	
Flax F+RF1d	3	1	1					2
Flax F+RF7d	5							

Abbreviations of the study groups are explained in Table 2.

**Table 4:** Occurrence of histological hyaluronan (HA; range 0–3 depending on the extent of reactivity) around bile ducts and in venous parts of the portal fields in the tundra vole livers according to dietary fat source and fasting/refeeding.

Study Group	Lard C	Lard F	Lard F+RF1d	Lard F+RF7d	Flax C	Flax F	Flax F+RF1d	Flax F+RF7d
**HA Bile Ducts**								
0	0	0	0	1	1	0	0	0
1	4	3	3	3	3	4	2	4
2	0	2	2	1	1	1	2	0
3	1	0	0	0	0	0	1	1
**HA Venous**								
0	0	1	0	1	1	0	0	0
1	3	3	3	3	3	4	2	4
2	2	1	2	1	1	1	2	0
3	0	0	0	0	0	0	1	1

Abbreviations of the study groups are explained in Table 2.

### 3.4. Fatty Acid Profiles of the Diets and Control Livers

**Figure 3:**
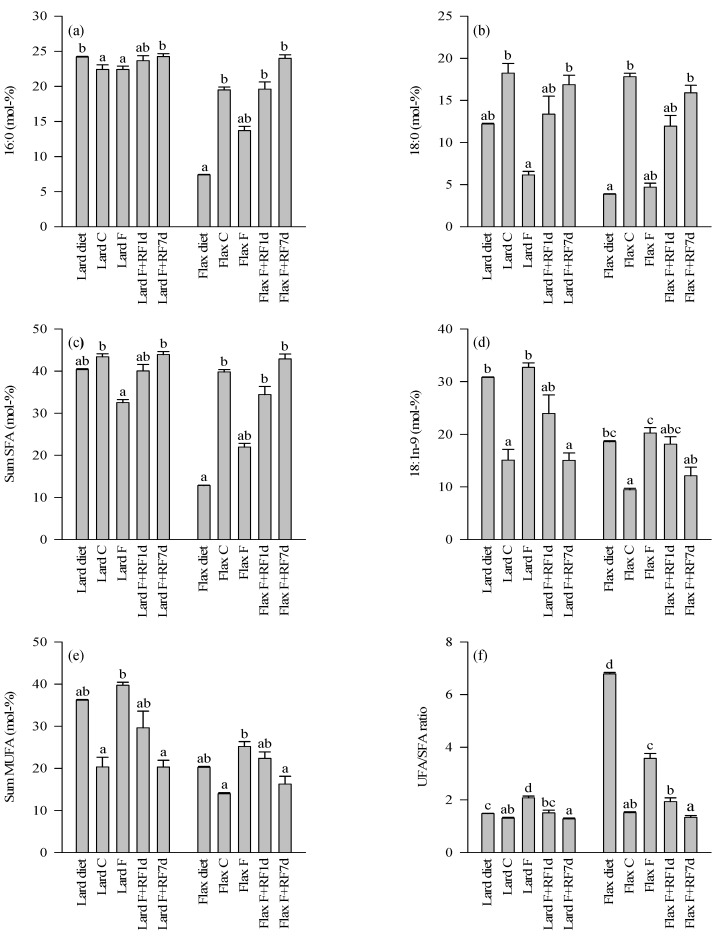
The percentages (mol-%) of selected saturated (SFA; **a**,**b**) and monounsaturated fatty acids (MUFA; **d**), fatty acid sums (**c**,**e**), and unsaturated (UFA)/SFA ratios (**f**) in the lard- or flaxseed oil-based diets and livers of the tundra voles fed these diets, fasted for18 h, and refed for 1 or 7 days after the 18-h fast (mean + SE). Means with no common letters are statistically different from each other within a diet (oneway ANOVA, Kruskal–Wallis ANOVA, *p* < 0.05). Abbreviations of the study groups are explained in Table 2.

**Figure 4:**
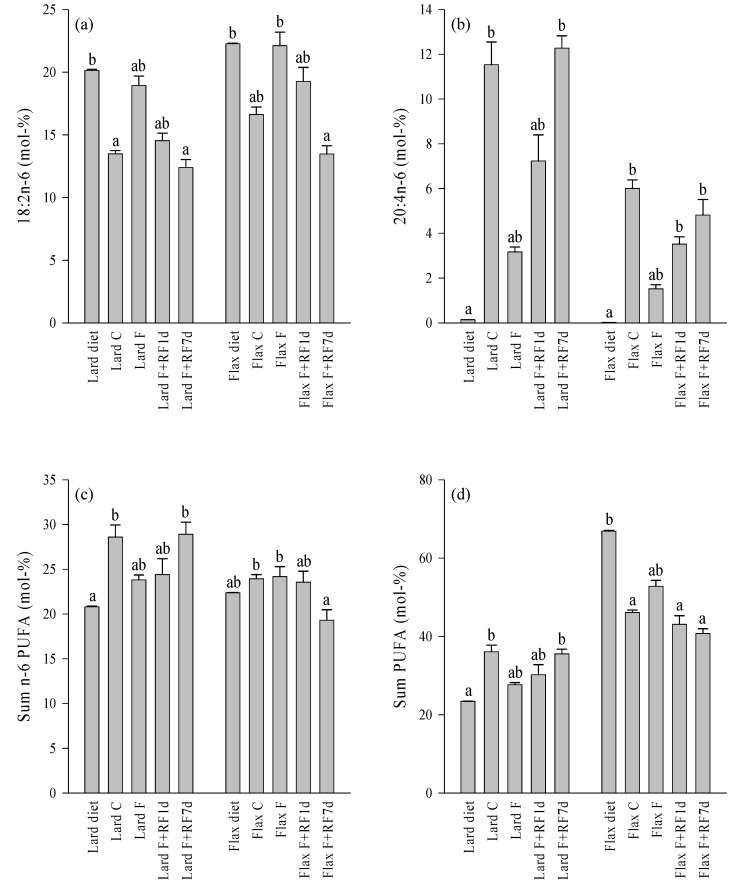
The percentages (mol-%) of selected *n*-6 polyunsaturated fatty acids (PUFA; **a**,**b**) and *n*-6 and total PUFA sums (**c**,**d**) in the lard- or flaxseed oil-based diets and livers of the tundra voles fed these diets, fasted for 18 h, and refed for 1 or 7 days after the 18-h fast (mean + SE). Means with no common letters are statistically different from each other within a diet (oneway ANOVA, Kruskal–Wallis ANOVA, *p* < 0.05). Abbreviations of the study groups are explained in Table 2.

**Figure 5:**
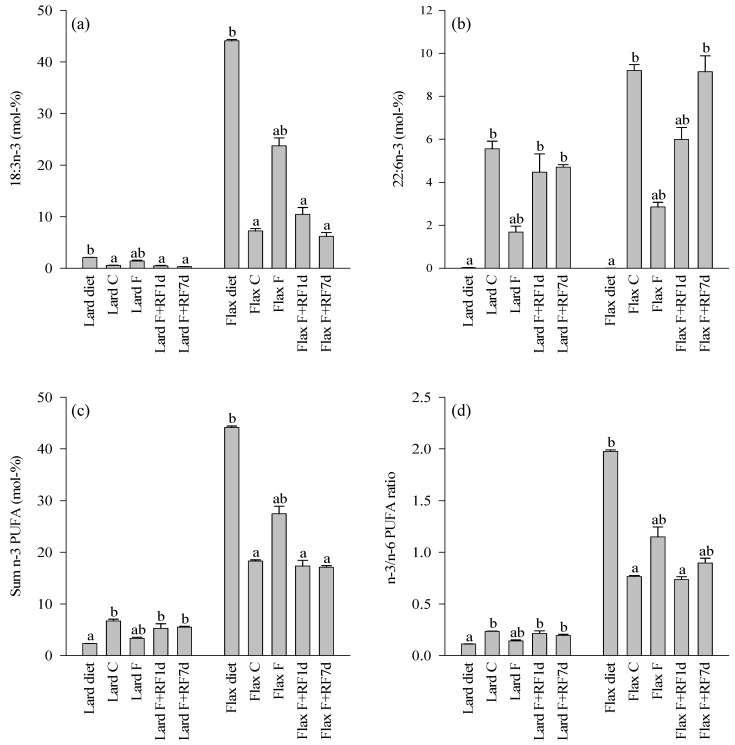
The percentages (mol-%) of selected *n*-3 polyunsaturated fatty acids (PUFA; **a**,**b**), *n*-3 PUFA sums (**c**), and *n*-3/*n*-6 PUFA ratios (**d**) in the lard- or flaxseed oil-based diets and livers of the tundra voles fed these diets, fasted for 18 h, and refed for 1 or 7 days after the 18-h fast (mean + SE). Means with no common letters are statistically different from each other within a diet (oneway ANOVA, Kruskal–Wallis ANOVA, *p* < 0.05). Abbreviations of the study groups are explained in Table 2.

The lard diet contained higher proportions of total SFA (mainly 14:0, 16:0, and 18:0) and MUFA (16:1*n*-7, 18:1*n*-9, 18:1*n*-7) and lower percentages of total *n*-6 and *n*-3 PUFA, especially the essential linoleic acid (LNA) and ALA, than the flaxseed oil diet (Figure 3, Figure 4 and Figure 5, Table S1). The proportions of particular *n*-6 (20:3*n*-6, ARA, 22:4*n*-6) and *n*-3 LCPUFA (EPA, 22:5*n*-3 [DPA*n*-3], DHA) were slightly higher in the lard diet. The *n*-3/*n*-6 PUFA and unsaturated FA (UFA)/SFA ratios were higher in the flaxseed oil diet.

The differences in the hepatic FA profiles of the fed control voles mostly followed the same pattern (Figure 3, Figure 4, and Figure 5, Table S1). The Lard C group had higher proportions of total SFA (mainly 16:0) and MUFA (18:1*n*-9) and lower percentages of total *n*-3 PUFA (ALA, EPA, DPA*n*-3, DHA) compared to the Flax C group. The LNA percentage was also lower in the Lard C livers but the *n*-6 PUFA sum was higher due to the increased levels of ARA and some other *n*-6 LCPUFA in the lard-fed voles. The *n*-3/*n*-6 PUFA and UFA/SFA ratios were higher in the Flax C group.

FA with efficient incorporation from the diet to the livers included 16:0 (Flax C only), 18:0, 20:3*n*-6, ARA, EPA (especially Flax C), 22:4*n*-6, DPA*n*-3, 22:5*n*-6 (especially Lard C), and DHA (Table S1). The proportions of 18:1*n*-9, LNA, and ALA were higher in the diets than in the tissues of the C groups.

### 3.5. Effects of Fasting on the Liver FA Profiles

The fasted groups of both dietary regimes showed decreases in the percentages of 18:0, total SFA, ARA, and DHA compared to their respective controls (Figure 3, Figure 4, and Figure 5, Table S1). Increased proportions were documented for 18:1*n*-9, total MUFA, LNA, and ALA as well as for the UFA/SFA ratios in the fasted animals with both dietary lipid sources. Several minor LCPUFA (20:3*n*-6, EPA, 22:4*n*-6, DPA*n*-3) decreased in proportion independent of the dietary history. There were also clear differences in the responses to fasting depending on the diet. The Flax F group showed decreased percentages of 16:0 while its proportions remained stable in the Lard F group compared to the fed controls. The *n*-6 PUFA sum decreased due to fasting in the Lard F voles but did not respond in the Flax F group. The total *n*-3 PUFA, total PUFA, and *n*-3/*n*-6 PUFA ratios increased in the Flax F group but the opposite was observed for the Lard F animals.

### 3.6. Effects of Refeeding on the Liver FA Profiles

The refed groups of both dietary fat sources showed increased percentages of 18:0, total SFA, and ARA after one day of realimentation but the values did not yet reach the control levels (Figure 3 and Figure 4, Table S1). 18:1*n*-9, total MUFA, and LNA reduced in proportions in a similar pattern, but the decreases tended to be clearer in the lard-fed animals. The percentages of ALA and the UFA/SFA ratios decreased close to the respective control levels but the *n*-6 PUFA sum did not respond in either dietary regime at this point (Figure 3, Figure 4 and Figure 5). 16:0 increased in proportion to the levels of the controls in the Flax F+RF1d group but remained quite stable in the refed lard voles. DHA increased in both dietary regimes; the *n*-3 PUFA sums and *n*-3/*n*-6 PUFA ratios increased to the control levels in the lard-fed animals and a similar pattern was observed in their decreases in the flaxseed oil group. The total PUFA percentage of the Lard F+RF1d voles did not change but that of the Flax F+RF1d group decreased close to the levels of their controls.

After seven days of refeeding, percentages of 18:0, total SFA, 18:1*n*-9, total MUFA, LNA, and ALA as well as the UFA/SFA ratios had returned close to or at the level of the respective control animals with both dietary fat sources (Figure 3, Figure 4 and Figure 5, Table S1). 16:0 of the Flax F+RF7d group increased further to the values of 16:0 in the lard-fed groups, which did not respond clearly to the experimental procedures. The *n*-6 PUFA sum of the Lard F+RF7d voles increased to the levels of their controls but the proportions of the Flax F+RF7d group decreased to a lower level than in their control group. The DHA percentages of the flaxseed oil-fed animals reached the control values by seven days of refeeding.

## 4. Discussion

Earlier evidence indicates that PUFA induce beneficial health effects compared to SFA and that *n*-3 PUFA have a greater cardioprotective effect than *n*-6 PUFA [38,39]. In addition to heart disease, *n*-3 PUFA from fish oil have been investigated for potentially favorable health effects on, e.g., rheumatoid arthritis, dementia, depression, and cancers [40,41]. Previous studies suggest that LC *n*-3 PUFA could also protect animals against body fat deposition [42] and fatty liver [43], while high-fat (60%) lard diets promote visceral obesity and steatosis [10,29]. In the present study, the dietary lipid source did not affect the appetite, weight gain, body or liver fat-% of the fed control voles. The fat content of the diets was 8%, and even though this was twice as high as that of the standard laboratory rodent chow fed previously to this vole strain, it did not induce liver fat accumulation. However, the mass of visible body fat reserves (mean approximately 4.9 g) was clearly higher compared to tundra voles fed the standard chow (<0.5 g; [25,26]), although it must be emphasized that the age of voles and the methodologies of fat mass measurements were not identical in these studies.

The FA profiles of the experimental diets were clearly reflected in the liver FA signatures of the voles as documented previously for other species [44,45]. The flaxseed oil-based diet contained more ALA and total *n*-3 PUFA with a higher *n*-3/*n*-6 PUFA ratio than the lard diet and these differences were detectable in the liver FA profiles of the fed voles. The lard-fed animals, on the other hand, had higher liver percentages of 16:0, total SFA, 18:1*n*-9, and total MUFA similar to their diet. However, the higher proportion of 18:0 in the lard diet did not translate into clear differences in the liver profiles possibly due to its desaturation to 18:1*n*-9 and chain-shortening to 16:0. The intakes of ARA, EPA, DHA, and other LCPUFA were very low with both feeds. In the lard diet, EPA, DPA*n*-3, and DHA were slightly higher in proportion but their liver percentages were elevated in the flaxseed oil-fed voles. This was expected, as flaxseed oil is the richest known source of ALA [6] that is subsequently metabolized to its desaturation and elongation products in a tissue-dependent manner.

Independent of the dietary fat source, fasting induced microscopically visible fatty liver without inflammation or fibrosis, see also [26]. The distribution of steatosis was mostly panacinar or azonal, thus resembling more closely the pediatric NAFLD than the zone 3 distribution found in adult patients [46]. The liver fat-% of the voles increased from <6% to 22% in 18 h without food independent of the preceding diet. This corresponds well to the liver fat-% of approximately 20% in a previous study on tundra voles that were fed the regular laboratory rodent chow and fasted for the same period of time [26]. The dietary lipids did not affect the rate of fasting-induced weight loss or the body fat-% and liver histology at the end of food deprivation.

Food deprivation modified the liver FA signatures to resemble more closely the dietary profiles. The fasted voles with the lard diet showed increased percentages of 18:1*n*-9, LNA, and ALA compared to their fed controls. The first two of these FA were present at high proportions in the lard-based diet and the percentages of all three, especially ALA, are known to decrease in vole adipose tissues due to fasting [26]. It is probable that these FA were released into the circulation by lipolysis from white (and possibly brown) adipose tissue, entered the liver, and were subsequently esterified with glycerol into TAG. This could lead to the increased liver fat-% in a situation with inadequate β-oxidation and/or formation and secretion of very-low-density lipoprotein [3,4]. Also the livers of the fasted flaxseed oil-fed voles accumulated FA that were both abundant in their diet and readily mobilized during fasting (*i.e.*, 18:1*n*-9, LNA, ALA).

It is known that *n*-3 LCPUFA and *n*-3/*n*-6 PUFA ratios are reduced in livers of NAFLD patients [5,7]. These changes were also observed in the lard-fed voles with fasting-induced fatty liver, whereas the flaxseed oil-fed animals showed reduced *n*-3 LCPUFA proportions but elevated *n*-3/*n*-6 PUFA ratios presumably due to increased influx of ALA to the liver from TAG hydrolysis in adipose tissue. The FA with reduced proportions in the livers of the fasted lard-fed animals included, e.g., 18:0, ARA, and DHA. These FA are all commonly enriched in phospholipids (PL) [47] of lipid bilayers, and their proportional decrease could be expected in the fasted voles, as when the amount of liver lipids increases, the relative proportions of FA rich in the TAG pool are elevated at the expense of FA prevalent in membrane PL [26]. In addition to 18:0, ARA, and DHA, also 16:0 showed a clear reduction in proportion during fasting in the flaxseed oil-fed voles. This was not observed in the lard-fed voles with stable 16:0 percentages during food deprivation.

According to the present results, the ALA-enriched diet, compared to the diet with a high SFA content, did not protect the voles from developing the fasting-induced fatty liver, nor did it ameliorate the severity of the hepatic fat accumulation. In previous studies, EPA+DHA protected the murine liver against steatosis [43] and EPA prevented the progression of steatosis induced by a Western-style diet in mice [8]. In reference to humans, it was documented that NAFLD patients can consume less fish and more meat than subjects with normal livers [12] and long-term EPA + DHA(+DPA*n*-3) supplementation promotes recovery from NAFLD [22,48]. While the reasons for the different findings are unknown, it is possible that the antisteatogenic effects of *n*-3 PUFA could be species-specific. It must be also emphasized that the present study utilized flaxseed oil high in ALA that is not equivalent to feeding fish oil high in EPA/DHA, even though the livers had accumulated these metabolites. In humans, the endogenous conversion of ALA to EPA and DHA is inefficient, but the rates have been suggested to be higher in laboratory rodents [49]. The tundra vole could have high Δ6-desaturase expression in the liver to achieve the notable LC *n*-3 PUFA percentages when fed flaxseed oil.

The different dietary lipids did not affect the appetite, weight gain, body fat-%, or liver histology during or after the one day- or seven day-refeeding periods that followed the 18 h of fasting. The relative liver weights were higher in three out of the four refed groups compared to the respective controls. The most likely explanations to this observation include the rapid increase in liver glycogen stores after fasting and residual hepatic lipids persisting in the organ during recovery even if the amounts were statistically insignificant. The liver fat-% and TAG concentrations returned close to the levels of the respective control groups as soon as after one day of refeeding. Although the liver fat-% was the same in the fasted voles of both dietary regimes, the liver TAG concentration was higher in the fasted flaxseed group. This could suggest a more severe fatty liver in the flaxseed voles at the start of refeeding, and perhaps for this reason their recovery did not differ from that of the lard-fed voles.

Liver recovery was also indicated by the gradual normalization of the FA signatures. After one day, the profiles were already approaching those of the controls and, after seven days, the proportions of several FA were at values similar to the fed animals. Notable exceptions to this were the increased 16:0 percentages and the reduced LNA proportions in the Flax F+RF7d group compared to the control levels. Elevated levels of 16:0 could result from, e.g., increased *de novo* lipogenesis and chain-shortening of 18:0 in the refed flaxseed voles. As *n*-3 PUFA upregulate the expression of genes encoding proteins involved in FA oxidation and downregulate those encoding proteins of lipid synthesis [20], their relative proportions in the liver can affect both the development of fatty liver and the recovery from steatosis. This was not observed in the voles, although previously long-term oral *n*-3 PUFA supplementation promoted recovery from NAFLD in humans [22,23] and, in rodents, *n*-3 PUFA had benefits on hepatic steatosis induced by a liver X receptor agonist or by CLA [9,50]. Our data illustrate that the potentially beneficial effects of *n*-3 PUFA on steatosis are not straightforward but can be species-specific. Furthermore, no correlation of HA staining with the histological findings of fatty liver was detected indicating that in the acute stage of fasting-induced hepatic injury or its recovery HA does not play a significant role in this species. Regarding the chain of pathological events from steatosis via chronic inflammation and accumulation of connective tissue to fibrosis and cirrhosis, our model did not show signs of progress beyond simple steatosis as evidenced by the leukocyte and HA results.

## 5. Conclusions

To summarize, (i) the lard-based diet did not induce body or liver fat accumulation in the voles compared to the flaxseed oil lipids. The dietary FA composition was clearly reflected in the hepatic FA profiles of the fed animals, and (ii) fasting modified the liver FA composition to resemble more closely the dietary profile. This probably results from the mobilization of dietarily abundant FA from adipose tissues, their transportation to the liver, and esterification into TAG. The ALA-enriched diet was not effective in the prevention of fatty liver, and (iii) HA staining visualized in the portal fields showed no significant relation with steatosis development. (iv) Flaxseed oil rich in ALA did not further accelerate the rapid recovery of the fasted voles from fatty liver.

## Supplementary Files

Supplementary File 1 Supplementary Information (DOCX, 34 KB)
