# Supplementary material for: Manifestations of Fasting-Induced Fatty Liver and Rapid Recovery from Steatosis in Voles Fed Lard or Flaxseed Oil Lipids
Source: Nutrients. 2013 Oct 22;5(10):4211–30. doi: 10.3390/nu5104211 (PMC3820070; doi:10.3390/nu5104211)
Supplement: Supplementary File 1 — Supplementary Information (DOCX, 34 KB) [file nutrients-05-04211-s001.docx]

**Supplementary Information**

**Table S1.** Fatty acid composition of the diets and livers of the tundra voles fed, fasted, or fasted and refed (mean ± SE).

| **Fatty Acid** | **Dietary Fat** | **Diet** | **Liver Fed Control** | **Liver Fasted** | **Liver Fasted + Refed for 1 Day** | **Liver Fasted + Refed for 7 Days** | ***P*** | |
| --- | --- | --- | --- | --- | --- | --- | --- | --- |
| 14:0 | Lard | 1.638 ± 0.005B | 0.513 ± 0.138AB | 1.465 ± 0.112B | 0.702 ± 0.150AB | 0.478 ± 0.117A | 1.3 × 10^−4^ | |
|  | Flax | 0.155 ± 0.003A * | 0.268 ± 0.016AB * | 0.967 ± 0.092B * | 0.554 ± 0.046B | 0.425 ± 0.074AB | 2.8 × 10^−5^ | |
| 16:0 | Lard | 24.174 ± 0.087B | 22.408 ± 0.633A | 22.401 ± 0.483A | 23.664 ± 0.696AB | 24.240 ± 0.417B | 0.013 | |
|  | Flax | 7.393 ± 0.059A * | 19.481 ± 0.417B * | 13.701 ± 0.599AB * | 19.594 ± 1.013B * | 23.990 ± 0.512B | 2.6 × 10^−5^ | |
| 18:0 | Lard | 12.178 ± 0.103AB | 18.241 ± 1.146B | 6.139 ± 0.438A | 13.360 ± 2.135AB | 16.863 ± 1.125B | 2.4 × 10^−4^ | |
|  | Flax | 3.853 ± 0.067A * | 17.805 ± 0.408B | 4.697 ± 0.468AB * | 11.934 ± 1.266AB | 15.890 ± 0.891B | 5.9 × 10^−5^ | |
| Total SFA | Lard | 40.356 ± 0.236AB | 43.398 ± 0.697B | 32.525 ± 0.721A | 40.036 ± 1.556AB | 43.931 ± 0.705B | 2.7 × 10^−4^ | |
|  | Flax | 12.835 ± 0.084A * | 39.791 ± 0.570B * | 21.963 ± 0.881AB * | 34.419 ± 1.915B * | 42.889 ± 1.177B | 2.9 × 10^−5^ | |
| 16:1*n*-7 | Lard | 1.758 ± 0.008AB | 1.056 ± 0.202A | 2.692 ± 0.150B | 1.278 ± 0.411AB | 0.960 ± 0.162A | 2.9 × 10^−4^ | |
|  | Flax | 0.112 ± 0.003A * | 0.675 ± 0.051AB | 2.059 ± 0.360B | 0.992 ± 0.127B | 0.889 ± 0.187AB | 5.5 × 10^−5^ | |
| 18:1*n*-9 | Lard | 30.792 ± 0.110B | 15.099 ± 2.051A | 32.707 ± 0.842B | 23.936 ± 3.545AB | 15.056 ± 1.397A | 3.8 × 10^−4^ | |
|  | Flax | 18.576 ± 0.189BC * | 9.451 ± 0.289A * | 20.239 ± 1.054C * | 18.119 ± 1.405ABC | 12.118 ± 1.639AB | 4.5 × 10^−4^ | |
| 18:1*n*-7 | Lard | 2.229 ± 0.011 | 2.435 ± 0.085 | 2.238 ± 0.063 | 2.319 ± 0.101 | 2.387 ± 0.073 | 0.157 | |
|  | Flax | 0.808 ± 0.010A * | 2.021 ± 0.127C * | 1.444 ± 0.079B * | 1.576 ± 0.087BC * | 1.742 ± 0.099BC * | 8.2 × 10^−5^ | |
| Total MUFA | Lard | 36.196 ± 0.134AB | 20.333 ± 2.299A | 39.729 ± 0.698B | 29.614 ± 3.985AB | 20.302 ± 1.616A | 2.2 × 10^−4^ | |
|  | Flax | 20.260 ± 0.185AB * | 13.965 ± 0.243A * | 25.198 ± 1.152B * | 22.366 ± 1.509AB | 16.247 ± 1.880A | 5.6 × 10^−4^ | |
| 18:2*n*-6 | Lard | 20.140 ± 0.085B | 13.482 ± 0.263A | 18.937 ± 0.752AB | 14.533 ± 0.600AB | 12.383 ± 0.657A | 8.4 × 10^−5^ | |
|  | Flax | 22.273 ± 0.042B * | 16.620 ± 0.598AB * | 22.121 ± 1.074B | 19.262 ± 1.119AB * | 13.469 ± 0.669A | 1.6 × 10^−4^ | |
| 18:3*n*-3 | Lard | 2.115 ± 0.010B | 0.534 ± 0.058A | 1.386 ± 0.175AB | 0.455 ± 0.093A | 0.301 ± 0.028A | 3.7 × 10^−5^ | |
|  | Flax | 44.093 ± 0.257B * | 7.220 ± 0.473A * | 23.734 ± 1.539AB * | 10.443 ± 1.330A * | 6.189 ± 0.736A * | 3.9 × 10^−5^ | |
| 20:3*n*-6 | Lard | 0.064 ± 0.002A | 0.707 ± 0.079B | 0.139 ± 0.004AB | 0.430 ± 0.103B | 0.677 ± 0.059B | 4.3 × 10^−5^ |  |
|  | Flax | 0.023 ± 0.001A * | 0.720 ± 0.082B | 0.118 ± 0.018AB | 0.364 ± 0.038AB | 0.534 ± 0.027B | 2.2 × 10^−5^ |  |
| 20:4*n*-6 | Lard | 0.141 ± 0.002A | 11.531 ± 1.017B | 3.167 ± 0.220AB | 7.230 ± 1.168AB | 12.275 ± 0.547B | 2.8 × 10^−5^ |  |
|  | Flax | 0.016 ± 0.001A * | 6.008 ± 0.378B * | 1.526 ± 0.179AB * | 3.514 ± 0.330B * | 4.813 ± 0.697B * | 3.1 × 10^−5^ |  |
| 20:5*n*-3 | Lard | 0.026 ± 0.002AB | 0.043 ± 0.004B | 0.015 ± 0.001A | 0.034 ± 0.011AB | 0.047 ± 0.006B | 0.0028 |  |
|  | Flax | 0.012 ± 0.002A * | 0.312 ± 0.039B * | 0.078 ± 0.014AB * | 0.145 ± 0.011AB * | 0.279 ± 0.015B * | 2.8 × 10^−5^ |  |

**Table S1.** *Cont.*

| 22:4*n*-6 | Lard | 0.064 ± 0.003A | 0.434 ± 0.021B | 0.179 ± 0.021AB | 0.261 ± 0.061AB | 0.406 ± 0.026B | 5.9 × 10^−5^ |
| --- | --- | --- | --- | --- | --- | --- | --- |
|  | Flax | 0.013 ± 0.002A * | 0.096 ± 0.010B * | 0.041 ± 0.006AB * | 0.056 ± 0.009AB * | 0.099 ± 0.009B * | 4.6 × 10^−5^ |
| 22:5*n*-6 | Lard | 0.013 ± 0.002A | 2.002 ± 0.242B | 0.697 ± 0.076AB | 1.464 ± 0.331B | 2.747 ± 0.335B | 6.1 × 10^−5^ |
|  | Flax | 0.009 ± 0.002A | 0.135 ± 0.049B * | 0.125 ± 0.024B * | 0.068 ± 0.008AB * | 0.096 ± 0.015B * | 2.7 × 10^−4^ |
| 22:5*n*-3 | Lard | 0.048 ± 0.004A | 0.431 ± 0.033B | 0.176 ± 0.013AB | 0.219 ± 0.063AB | 0.295 ± 0.018B | 5.3 × 10^−5^ |
|  | Flax | 0.007 ± 0.001A * | 1.007 ± 0.036B * | 0.547 ± 0.073AB * | 0.486 ± 0.042AB * | 1.066 ± 0.057B * | 3.4 × 10^−5^ |
| 22:6*n*-3 | Lard | 0.026 ± 0.005A | 5.559 ± 0.351B | 1.684 ± 0.274AB | 4.467 ± 0.856B | 4.700 ± 0.122B | 5.6 × 10^−5^ |
|  | Flax | 0.005 ± 0.001A * | 9.201 ± 0.271B * | 2.849 ± 0.221AB * | 5.988 ± 0.563AB | 9.141 ± 0.746B * | 2.6 × 10^−5^ |
| Total PUFA | Lard | 23.389 ± 0.103A | 36.066 ± 1.690B | 27.676 ± 0.536AB | 30.208 ± 2.581AB | 35.538 ± 1.200B | 2.7 × 10^−4^ |
|  | Flax | 66.863 ± 0.219B * | 46.089 ± 0.644A * | 52.780 ± 1.521AB * | 43.077 ± 2.183A * | 40.725 ± 1.236A * | 4.2 × 10^−5^ |
| Total *n*-3 PUFA | Lard | 2.309 ± 0.013A | 6.695 ± 0.335B | 3.327 ± 0.195AB | 5.258 ± 0.882B | 5.482 ± 0.135B | 6.3 × 10^−5^ |
|  | Flax | 44.165 ± 0.256B * | 18.288 ± 0.233A * | 27.439 ± 1.485AB * | 17.323 ± 1.097A * | 17.102 ± 0.289A * | 4.4 × 10^−5^ |
| Total *n*-6 PUFA | Lard | 20.793 ± 0.088A | 28.596 ± 1.348B | 23.823 ± 0.529AB | 24.401 ± 1.771AB | 28.905 ± 1.341B | 4.4 × 10^−4^ |
|  | Flax | 22.367 ± 0.041AB * | 23.939 ± 0.462B * | 24.179 ± 1.117B | 23.566 ± 1.220AB | 19.308 ± 1.170A * | 0.00518 |
| *n*-3/*n*-6 PUFA ratio | Lard | 0.111 ± <0.001A | 0.234 ± 0.002B | 0.140 ± 0.010AB | 0.212 ± 0.026B | 0.192 ± 0.012B | 4.7 × 10^−5^ |
|  | Flax | 1.975 ± 0.015B * | 0.765 ± 0.010A * | 1.149 ± 0.095AB * | 0.735 ± 0.027A * | 0.896 ± 0.045AB * | 4.5 × 10^−5^ |
| UFA/SFA ratio | Lard | 1.477 ± 0.015C | 1.302 ± 0.037AB | 2.078 ± 0.068D | 1.510 ± 0.099BC | 1.273 ± 0.037A | 2.7 × 10^−4^ |
|  | Flax | 6.791 ± 0.051D * | 1.511 ± 0.036AB * | 3.580 ± 0.183C * | 1.933 ± 0.145B * | 1.336 ± 0.068A | 2.9 × 10^−5^ |

Means with no common letters are statistically different from each other within a row (oneway ANOVA, Kruskal–Wallis ANOVA, *p* < 0.05), * = difference between the diets or dietary groups within a column (*t-*test, Mann–Whitney U test, *p* < 0.05), SFA = saturated fatty acids, MUFA = monounsaturated fatty acids, PUFA = polyunsaturated fatty acids, UFA = unsaturated fatty acids (MUFA + PUFA).
